# Precision nutrition in companion animals: a response-based framework linking dietary strategies, metabolic function, and measurable clinical outcomes

**DOI:** 10.3389/fvets.2026.1914947

**Published:** 2026-10-21

**Authors:** Xinfang Du, Han Zhang, Weipeng Tian, Jinfa Chen, Min Wen, Wenlu Fan

**Affiliations:** 1 Pet Nutrition Research Center, Gambol Pet Food Group Co., Ltd., Shanghai, China; 2 Shandong Key Laboratory of Applied Technology for Protein and Peptide Drugs, Institute of Biopharmaceutical Research, Liaocheng University, Liaocheng, China

**Keywords:** artificial intelligence, companion animals, disease management, gut microbiome, multi-omics, precision nutrition

## Abstract

Population-level nutrient recommendations provide the foundation for complete and balanced diets in dogs and cats, but they do not fully account for inter-individual variation in nutritional requirements, metabolic phenotypes, and dietary responses. Precision nutrition provides a response-guided framework that integrates individual characteristics with monitored dietary responses to refine nutritional management through repeated reassessment. This review examines the conceptual framework, host determinants, clinical applications, and translational challenges of precision nutrition in companion animals. Obesity, chronic enteropathy, chronic kidney disease, and urolithiasis are discussed as representative clinical models in which dietary interventions can be linked to measurable outcomes and adjusted according to individual response. Current clinical implementation is informed primarily by phenotypic assessment, dietary history, routine clinical and biochemical measures, and longitudinal monitoring, with biomarkers such as serum phosphate providing direct guidance in specific clinical contexts. Microbiome and metabolomic profiles, selected molecular biomarkers, and digital monitoring tools, such as smart feeders and activity monitors, may provide complementary information, whereas multi-omics integration and AI-assisted prediction remain largely exploratory. Further progress will require standardized species-specific datasets, prospective validation of predictive biomarkers, reliable digital measurements, and externally validated, interpretable models that demonstrate added value beyond established clinical assessment. Overall, precision nutrition represents an iterative, response-guided framework in which individual characteristics and monitored outcomes inform dietary selection and adjustment over time.

## 1 Introduction

Dogs and cats are increasingly receiving long-term veterinary care, in which nutritional management plays a key role in maintaining health, supporting treatment, and reducing the risk of disease progression and recurrence. Chronic conditions such as obesity, chronic enteropathy, chronic kidney disease (CKD), and lower urinary tract disorders, particularly urolithiasis, are frequently encountered in small animal practice and often require sustained nutritional management (1–5). At the same time, long-term dietary exposure and feeding practices may also contribute to the development of some chronic conditions. However, it remains unclear which dietary characteristics, including nutrient composition and intake, food format, moisture content, feeding patterns, and processing-related characteristics, are most relevant to disease risk (6–8). Beyond these uncertainties in disease risk, animals with similar clinical conditions or receiving similar dietary recommendations may exhibit different nutritional responses, reflecting variation in biological characteristics and dietary adaptation. For example, obese dogs undergoing weight-management programs may show considerable differences in weight-loss outcomes despite receiving controlled dietary interventions, highlighting the limitations of relying solely on standardized feeding strategies (9, 10). Such response heterogeneity illustrates the need to consider individual variation when nutritional strategies are selected, evaluated, and adjusted over time.

Population-level nutrient recommendations are essential for formulating nutritionally adequate and balanced diets for dogs and cats (11), but they do not capture the full extent of inter-individual variation in nutrient requirements, metabolic phenotypes, or responses to the same diet. This variation is shaped by interacting host-related, clinical, and environmental factors, including species-specific physiology, genetic background, life stage, body composition, activity level, health status, feeding behavior, and gut microbiome composition (12–15). Studies in companion animals have demonstrated that these biological factors can influence nutritional outcomes. For example, genetic variation affecting appetite regulation has been associated with differences in body weight and feeding behavior in dogs, while variation in gut microbiome composition and microbial metabolites has been linked to differences in dietary tolerance, metabolic status, and nutrition-responsive conditions (15–18). Precision nutrition provides a framework for refining conventional feeding recommendations by integrating host characteristics, clinical indicators, dietary history, microbiome profiles, omics-derived information, and longitudinal response data to guide more targeted and adaptive nutritional management (12–14).

The concept of precision nutrition has been widely discussed in human nutrition and precision medicine, where individualized dietary recommendations and predictive models have shown potential to improve intervention outcomes (12). In companion animals, this field remains at an earlier translational stage, although advances in genomics, metabolomics, microbiome profiling, artificial intelligence (AI), wearable devices, smart feeding systems, and digital health platforms have created new opportunities for individualized nutritional assessment and management (14, 19, 20). However, the current clinical applicability of these approaches to nutritional management in dogs and cats varies considerably. Conventional assessments, including body condition evaluation, dietary history, clinical indicators, fecal characteristics, and monitoring of dietary response, remain the practical foundation of nutritional decision-making in dogs and cats (21). Omics-based and digital approaches currently play a more supportive role in biological characterization, risk assessment, and the future prediction of dietary responses (14, 19, 20). At the same time, precision nutrition in dogs and cats should be developed within a species-appropriate framework rather than directly adopting human nutrition models. Cats are obligate carnivores with distinctive nutritional requirements and metabolic adaptations. In addition to their specific requirements for taurine, arginine, and preformed vitamin A, cats exhibit sustained gluconeogenic activity and limited metabolic adaptation to reduced protein intake, reflecting their evolutionary dependence on protein-rich diets (22, 23). In contrast, dogs exhibit a more adaptable carnivore metabolic profile and greater adaptability to different macronutrient compositions, while also showing substantial variation in body size, digestive physiology, energy expenditure, and breed-associated disease risk (24). These physiological differences influence nutritional requirements and dietary responses, highlighting the need to integrate species-specific biology with measurable outcomes when developing precision nutrition strategies for companion animals.

To address these issues, this review synthesizes current evidence on precision nutrition in dogs and cats from conceptual, biological, clinical, and translational perspectives. It first defines the scope of precision nutrition and examines host-related determinants that shape nutritional requirements, disease risk, and dietary response. The clinical applications are then discussed using obesity, chronic enteropathy, chronic kidney disease and lower urinary tract disease, with a focus on urolithiasis, as representative contexts in which dietary strategies can be linked to measurable endpoints. Finally, the review considers key translational challenges, including biomarker validation, multi-omics integration, artificial intelligence-assisted decision support, data standardization, and feedback-guided dietary management. By integrating mechanistic evidence, clinical nutrition, and implementation considerations, this review aims to clarify how precision nutrition may complement and refine population-level feeding recommendations through more individualized, response-guided nutritional management in dogs and cats.

## 2 Conceptual framework of precision nutrition in companion animals

### 2.1 Precision nutrition: definition and scope in companion animals

Precision nutrition is an emerging nutritional approach in which measurable characteristics of the animal are used to guide dietary decisions, and the resulting response is evaluated using predefined outcomes. In companion animals, a precision nutrition intervention should integrate three fundamental components: characterization of individual nutritional and clinical phenotypes, selection of a dietary strategy informed by these characteristics, and longitudinal evaluation of predefined outcomes to support response-guided dietary adjustment. The information supporting this framework encompasses clinical phenotypes, dietary exposure, host biological characteristics, metabolic status, gut microbiome features, and longitudinal response data (12, 13, 25, 26). In dogs and cats, this framework should account for species-specific nutritional physiology, including the distinctive metabolic adaptations of cats and the substantial variation in digestive physiology among dogs (22–24). This framework is summarized in Figure 1, which illustrates how population-level nutritional foundations can be refined through individualized evidence layers and response-based dietary management.

**Figure 1:**
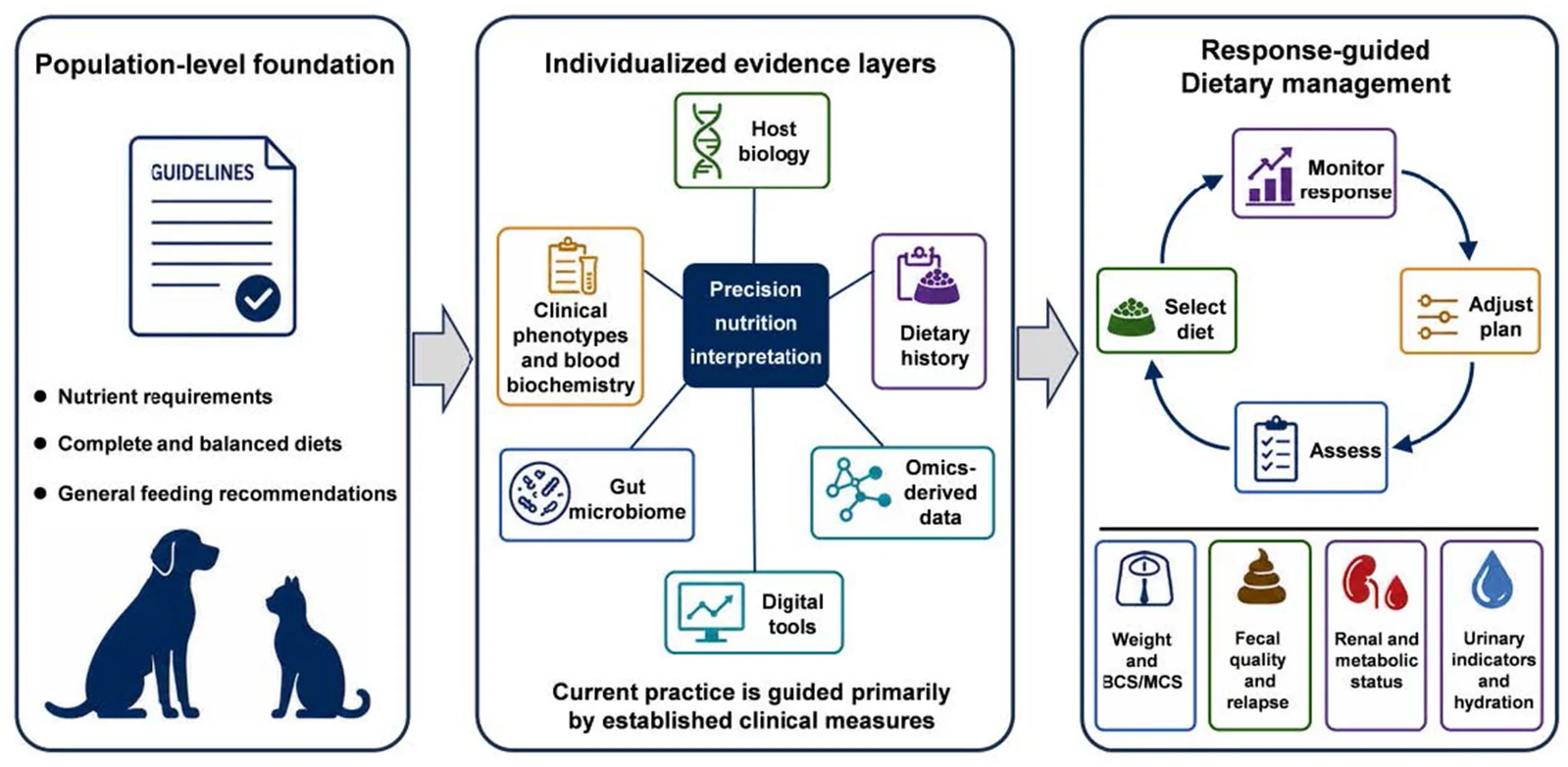
Overall framework of precision nutrition in dogs and cats. Precision nutrition builds on population-level feeding recommendations and refines them through individualized evidence layers and response-guided dietary management. Clinically accessible measures, including host biology, clinical phenotypes, blood biochemistry, and dietary history, currently provide the most practical basis for nutritional decision-making, whereas the gut microbiome, omics, and digital tools may further inform interpretation when they provide actionable and interpretable information. Dietary management is guided through an iterative cycle of diet selection, response monitoring, plan adjustment, and reassessment, with clinically actionable indicators such as weight and body or muscle condition, fecal quality and relapse, renal and metabolic status, and urinary indicators or hydration status used to evaluate outcomes. BCS, body condition score; MCS, muscle condition score.

Precision nutrition overlaps with personalized nutrition, but is distinguished by the use of measurable biological or clinical variation to guide dietary assessment, intervention, and follow-up within an evidence-informed framework. Personalized nutrition may include advice, products or services tailored to individual characteristics, behaviors, preferences or goals (12), whereas precision nutrition requires stronger emphasis on response prediction, biomarker-informed stratification, longitudinal monitoring and adjustment of dietary strategies (13, 25). Therapeutic diets, functional ingredients and other targeted dietary interventions may be incorporated within a precision nutrition framework, but their use alone does not necessarily constitute precision nutrition. Their role within such a framework depends on whether individual-specific biological, clinical or dietary information is used to inform dietary selection and whether measurable responses are subsequently evaluated to guide nutritional management (12, 27, 28). Accordingly, precision nutrition should be viewed as an extension of established clinical nutrition that integrates individual-specific information and response assessment into nutritional decision-making, rather than as a replacement for conventional dietary management.

### 2.2 Evidence inputs for precision nutrition

The data inputs relevant to precision nutrition differ in their current clinical applicability and contribution to nutritional decisions. In current veterinary nutrition practice, clinical phenotyping, dietary history and exposure, and measurable response outcomes provide the core information for nutritional assessment and longitudinal adjustment because they can be repeatedly evaluated and directly linked to dietary decisions (21, 27, 28). Biochemical measures, host-related characteristics, and digital monitoring may provide additional information when relevant to a specific nutritional objective. In contrast, omics-derived data may provide mechanistic insight and support future risk assessment or response prediction, but their routine application requires further evidence of analytical validity and clinical utility in dogs and cats (14, 16, 25).

Omics technologies provide complementary information for characterizing genetic susceptibility, molecular regulation, metabolic phenotypes, and host–microbiome interactions. Host-oriented approaches include genomics and epigenomics, transcriptomics, proteomics, metabolomics, lipidomics and phenomics, whereas microbiome-oriented approaches include metagenomics, metatranscriptomics, metaproteomics and microbial metabolomics (14, 29). More specialized systems-level approaches, such as exposomics, fluxomics, single-cell omics and spatial omics, may further characterize environmental, pathway-level and tissue- or cell-specific responses (30–33), although their application to precision nutrition in dogs and cats remains exploratory. In companion animals, omics-based studies have identified candidate features associated with appetite and body-weight regulation (16), inherited metabolic or urinary risk (34, 35), obesity-related metabolic phenotypes (10, 36), and diet-responsive microbial and metabolite changes in chronic enteropathy (18, 37). However, most omics-derived biomarkers in dogs and cats remain exploratory. Their use in routine nutritional management requires evidence that they add predictive or clinical value beyond conventional phenotypic and clinical assessment.

The gut microbiome is increasingly recognized as a relevant data layer in precision nutrition because it reflects interactions among diet, host physiology and health status, although its interpretation remains methodologically challenging. Microbial composition in dogs and cats varies with age (38, 39), breed (24, 40, 41), diet (15, 38), medication exposure, particularly antimicrobial treatment (42), and physiological and disease states (17, 18, 43). A recent review of the canine gut microbiome further emphasizes its roles in digestion, immune regulation, energy metabolism, and host–environment interactions, supporting the relevance of microbiome-based information to nutritional assessment while highlighting the need for standardized methodology and interpretation (44). Dysbiosis and altered microbial metabolism have been reported in obesity, chronic enteropathy and other nutrition-responsive conditions, suggesting that microbiome-derived information may help refine future dietary response assessment and individualized nutritional management (45, 46). Metabolomic profiling may provide a complementary layer for characterizing diet–microbiome–host interactions by capturing diet-responsive microbial and host metabolites, although integrated microbiome–metabolome analyses and validated metabolite markers remain limited in dogs and cats (47). However, microbiome findings are sensitive to sampling procedures, recent diet, medication exposure, sequencing platform, bioinformatic pipeline, and reference database (48). Therefore, microbiome-informed interpretation should be integrated with clinical endpoints rather than used as a stand-alone basis for dietary prescription.

Artificial intelligence and machine learning may support the integration of high-dimensional datasets for feature selection, risk prediction, responder classification, and individualized nutritional recommendations (20, 29, 49). However, their application in companion animal nutrition remains limited by small sample sizes, heterogeneous study designs, insufficient longitudinal datasets, and limited external validation (50, 51). Therefore, data inputs in precision nutrition can be viewed along a continuum from clinically established indicators to exploratory biomarkers. Future studies should determine whether specific data inputs improve prediction, dietary response assessment, or clinical outcomes beyond established nutritional assessment, and whether they can be translated into interpretable and monitorable dietary recommendations for dogs and cats. Major information domains relevant to precision nutrition differ in their current clinical applicability, degree of methodological validation, and clinical utility, as summarized in Table 1.

**Table 1:** Major data layers supporting precision nutrition in dogs and cats.

Data layer	Representative measures or data	Current status and evidence base	Clinical utility	Representative references
Clinical phenotypes and routine assessment	Body weight, body condition score, muscle condition score, fecal quality, urinary signs, disease history and previous dietary response	Established. Routinely assessed using clinically interpretable and repeatable measures	Provides the primary basis for phenotyping, selection of monitoring endpoints and response-guided dietary adjustment	(21, 27, 96)
Blood-based clinical indicators	Hematology, serum biochemistry, glucose, serum proteins, electrolytes, renal and hepatic measures, and selected endocrine or inflammatory markers	Established but context dependent. Laboratory methods are widely available, whereas interpretation depends on disease status and clinical context	Characterizes metabolic and organ status and supports monitoring of nutritional response and safety	(5, 21, 112, 142)
Dietary history and feeding practices	Diet type and composition, feeding amount, food format, treats, water intake, feeding frequency and owner adherence	Established. Readily obtainable, although accuracy depends on dietary records, owner recall and adherence	Clarifies actual dietary intake and feeding practices and identifies modifiable dietary factors	(21, 27, 96)
Host characteristics	Species, breed and body size, life stage, reproductive status and activity level	Established. Routinely available, although their nutritional relevance varies with clinical context	Helps define nutritional priorities, risk patterns and monitoring needs	(11, 21, 24, 73)
Gut microbiome and microbial metabolites	Microbial composition, dysbiosis indices, microbial pathways, short-chain fatty acids, bile acids and microbial co-metabolites	Supportive/emerging. Analytical methods are available, but results are sensitive to sampling, diet, medication exposure and analytical workflow, and clinical validation remains limited	May complement assessment of gastrointestinal phenotype, dietary response and host–diet–microbiome interactions, but is not sufficient for stand-alone diet selection	(15, 18, 44–48)
Metabolomic and other omics-derived profiles	Genomic information; serum, plasma, urine or fecal metabolomics; proteomic and lipidomic profiles; candidate molecular signatures	Emerging. Analytical platforms are well-developed, but prospective validation, cross-platform comparability and clinically validated thresholds remain limited	May support molecular and metabolic phenotyping, mechanism discovery and identification of candidate predictors or response biomarkers	(14, 29, 36, 47, 157)
Digital monitoring tools	Smart feeders, connected scales, activity monitors and other home-monitoring devices	Supportive/emerging. Increasingly available, although measurement accuracy, data completeness and interoperability vary among devices	Enables repeated home-based monitoring of food intake, body weight, activity and adherence	(160, 161)
AI- and machine-learning approaches	Multimodal data integration, responder classification, risk stratification and dietary-response prediction	Exploratory. Current applications are largely study-specific, with limited external validation and prospective clinical testing	May support data integration, pattern recognition and future response prediction, but should not replace validated clinical assessment	(159, 162, 163)

### 2.3 Nutritional decision and feedback

Precision nutrition should be implemented as an iterative decision process rather than as a one-time data-driven recommendation. After clinical, dietary, and biological information has been interpreted, the next step is to define the nutritional objective and select a dietary strategy that can be evaluated over time. In dogs and cats, such objectives may include adjusting energy intake, preserving lean mass, improving fecal quality, modifying dietary fiber or protein sources, increasing water intake, reducing urinary supersaturation, selecting a therapeutic diet or avoiding specific dietary antigens (21, 28). The choice of strategy should be based on the animal's clinical phenotype, dietary history, disease context, and likelihood of owner adherence, rather than solely on biomarker results (21, 28).

Feedback-based evaluation is a key component of this framework, and each nutritional strategy should be evaluated using predefined, measurable outcomes that are relevant to the nutritional goal. The selection and interpretation of these endpoints should be guided by host-related determinants, disease context, and the nutritional goal. For example, weight-management plans may be evaluated using body weight, body condition, muscle condition, food intake and adherence; gastrointestinal dietary interventions should be assessed through fecal quality, appetite, tolerance, body weight and relapse control; and urinary strategies should be monitored using urine dilution, urinary pH, stone composition, recurrence risk and other urinary indicators where available (21, 27, 28). In this way, precision nutrition differs from simple diet customization or product matching: the dietary plan is refined according to monitored response rather than fixed after the initial assessment (12, 25).

Candidate biomarkers, omics-derived profiles, and algorithm-assisted models may strengthen this decision process by improving risk assessment, identifying likely responders, and predicting dietary response. However, these tools should be incorporated only when they provide interpretable and clinically useful information beyond established nutritional assessment. Their value should be evaluated through controlled feeding trials, longitudinal studies and routine veterinary practice, with attention to feasibility, owner adherence and measurable health outcomes (14, 20, 25). Accordingly, feedback should be viewed as more than post-intervention observation; it is the process through which dietary strategies are evaluated and adjusted over time.

## 3 Host determinants of precision nutrition in dogs and cats

Host-related factors provide the biological and practical basis for precision nutrition in dogs and cats. These factors include genetic background, breed-related variation, life stage, reproductive status, body condition, lifestyle, activity level, and owner-driven feeding practices. These factors should inform nutritional risk assessment, monitoring priorities and individualized dietary adjustment, rather than being used as stand-alone criteria for nutritional management. These host-related factors are best interpreted as part of an interacting host–diet–microbiome–clinical system that shapes dietary response phenotypes, rather than as isolated criteria for diet selection (Figure 2).

**Figure 2:**
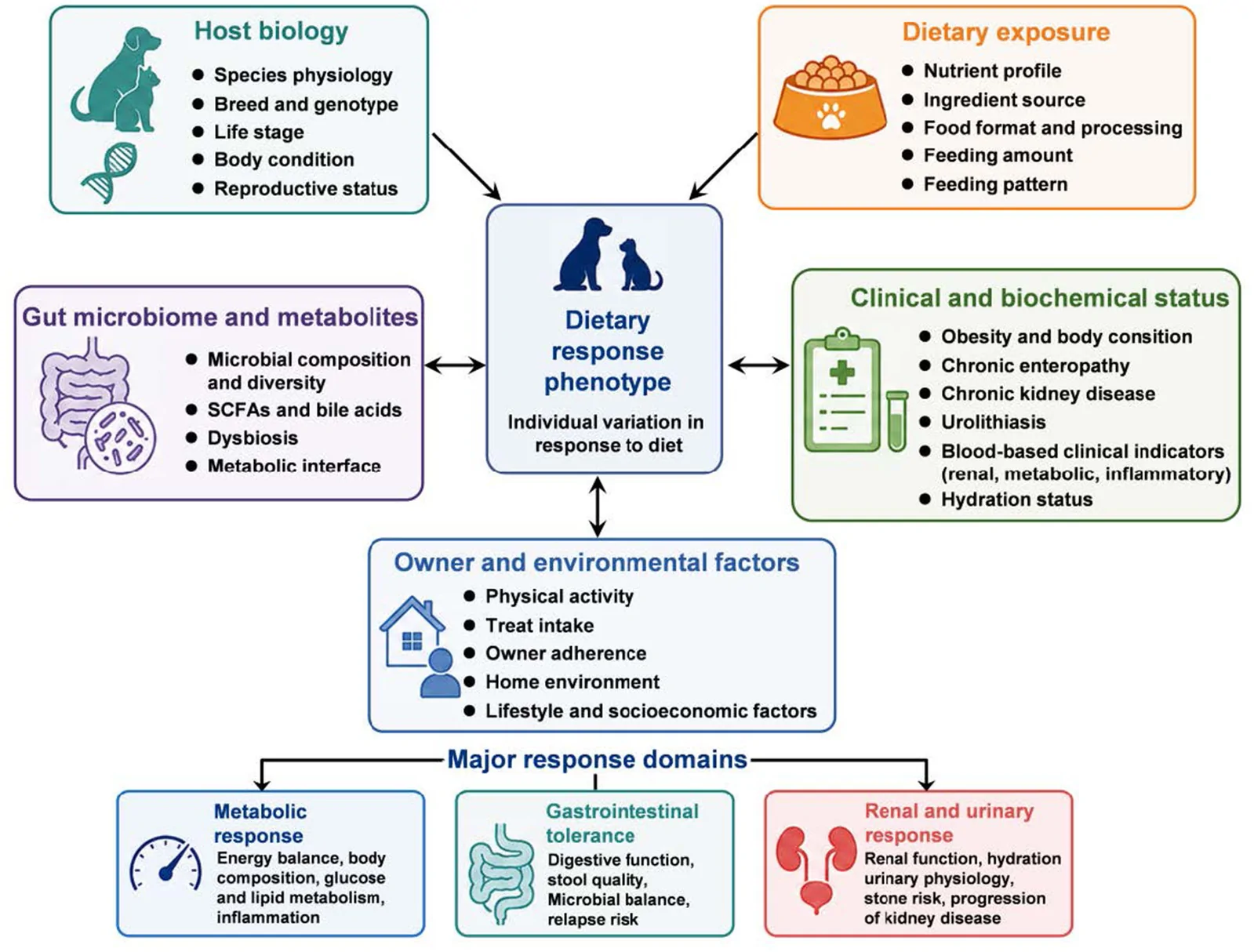
Interacting determinants of dietary response in dogs and cats. Dietary response phenotypes in dogs and cats arise from interactions among host biology, dietary exposure, gut microbiome and metabolites, clinical and biochemical status, and owner and environmental factors. These determinants influence major response domains, including metabolic response, gastrointestinal tolerance, and renal and urinary response, and together provide the biological and practical context for individualized nutritional assessment and response-guided dietary management. SCFAs, short-chain fatty acids.

### 3.1 Genetic background and breed variation

Genetic background and breed variation are important sources of inter-individual differences in nutritional requirements, dietary tolerance, and disease risk in dogs and cats. In dogs, selective breeding has produced substantial diversity in body size, digestive physiology, metabolic phenotype, gut microbiome composition and disease susceptibility. These differences may influence energy requirements, nutrient utilization, fecal quality, dietary tolerance and the likelihood of conditions requiring nutritional management. Comparative studies suggest that canine body-size categories and breeds may differ in digestive physiology and gut microbiome composition (24, 41).

Evidence related to genetic background, breed variation, and body size should be interpreted in relation to the specific phenotype and outcome assessed in each study. In dogs, the pro-opiomelanocortin (POMC) deletion has been associated with body weight and appetite regulation in obesity-prone Labrador Retrievers, suggesting potential relevance to satiety-oriented feeding strategies and weight-management monitoring (16). In Beagle dogs, specific variants in melanocortin 4 receptor (MC4R) have been associated with body-weight variation (52). In Labrador Retrievers, a genome-wide association study identified DENN domain containing 1B (DENND1B) as an obesity-associated locus, and functional analyses further showed that DENND1B regulates MC4R trafficking and signaling (53). These findings indicate that genetic variation may influence obesity-related phenotypes through different components of appetite and energy-homeostasis pathways. In cats, epidemiological studies have reported breed differences in diabetes risk, including increased risk in Burmese cats, and genome-wide association analysis has identified candidate genes for diabetes susceptibility in non-obese cats (35, 54, 55). Inherited disorders such as cystinuria linked to solute carrier family 7 member 9 (SLC7A9) variants further illustrate that genetic variation may influence specific urinary or metabolic risk phenotypes relevant to nutritional monitoring (34). Similarly, breed- or population-level variation in urolithiasis patterns may help identify animals that require targeted urinary monitoring; however, dietary prevention should be guided by stone composition, urine pH, urine specific gravity, infection status, relative supersaturation (RSS) where available, and recurrence history rather than breed alone (27, 56).

Breed and genotype should not be treated as stand-alone determinants of nutritional management in precision nutrition. Instead, they should be used to identify risk patterns that may support targeted monitoring, earlier intervention, or more individualized follow-up. Importantly, dogs of the same breed may still differ genetically because geographically or reproductively isolated populations can develop distinct genetic backgrounds. Geographic isolation and breeding practices can generate differentiated subpopulations, whereas increasing international exchange of breeding animals may introduce gene flow between previously more isolated populations; conversely, restricted breeding and repeated use of popular sires can reduce effective genetic diversity (57, 58). In a precision nutrition framework, breed information and genetic markers should be interpreted together with population or lineage background, clinical phenotype, dietary exposure, owner-reported feeding behavior, and monitored response when dietary recommendations are developed.

### 3.2 Life stage, reproductive status and body condition

Life stage substantially shapes nutritional priorities in dogs and cats. Early life, weaning, adulthood, and aging are accompanied by changes in growth, metabolism, immune function, gastrointestinal physiology, and disease susceptibility that may influence energy requirements, nutrient utilization, and the relative importance of specific dietary strategies. Accordingly, nutritional management should be adjusted throughout the life course based on body condition, activity level, functional status, comorbidities, and monitored response (11, 21).

Early life represents a critical window for growth, immune development, and gut microbiome maturation. Nutritional management during this period should first ensure adequate energy, high-quality protein, and essential nutrients to support normal growth and physiological development (59, 60). Kitten studies and broader mammalian research indicate that early-life nutrition may support not only nutrient adequacy, but also gut microbiome development, immune maturation and metabolic regulation (61, 62). Maternal colostrum and milk provide a biological model for early-life nutritional support because they supply macronutrients and bioactive components, including immunoglobulins, lactoferrin, oligosaccharides, bioactive lipids, antimicrobial peptides and growth-related factors involved in passive immune transfer, intestinal maturation, microbial colonization and mucosal defense (63, 64). The weaning transition is another sensitive period, during which dynamic changes in kitten gut microbiota, serum metabolites and immune-related indicators support the need for careful nutritional assessment (62). Beyond supporting immediate growth and development, dietary and environmental exposures during early life may also be associated with health outcomes later in life. Canine nutritional epidemiology studies spanning gestation, lactation, puppyhood and adolescence have examined different feeding patterns together with environmental and host-related factors, and have reported associations with subsequent risks of owner-reported atopic dermatitis, chronic enteropathy and otitis (65–68). These findings suggest that both the type and timing of nutritional exposure may be relevant to long-term health. Candidate early-life nutritional interventions, including complex probiotics, compound polysaccharides and bovine colostrum, have been reported to modulate gut microbiota, fecal characteristics, constipation-related outcomes, immune function and antioxidant status in kittens (69–71). These approaches may provide useful tools for response-guided early-life nutrition when their use is aligned with growth trajectory, fecal quality, maternal lactation status, immune status, and clinical follow-up.

In adulthood, nutritional management shifts from supporting growth to maintaining optimal body condition, lean body mass, metabolic stability, and long-term health. Maintenance energy requirements vary with sex, neutering status, body condition, activity level and underlying health conditions, and predictive models in adult dogs suggest that actual requirements may differ from conventional estimates (72). Regular nutritional assessment throughout adulthood should therefore consider body condition, muscle condition, dietary intake, activity level, and health status, with feeding strategies adjusted according to longitudinal changes and monitored response (21). Nutritional management in adulthood should focus on individualized energy allocation, body-condition assessment, appetite or satiety management, and adjustment according to activity level and underlying health conditions. Neutering is an important consideration because it may alter appetite regulation, reduce energy requirements, and increase the risk of weight gain in dogs and cats (73, 74). Post-neutering feeding plans should emphasize body-condition-based energy adjustment and satiety management, with feeding amounts revised according to changes in body weight and body condition rather than fixed recommendations. In this context, body weight, body condition score (BCS), and muscle condition score are clinically actionable phenotypes because they can be repeatedly assessed and directly linked to dietary adjustment (75, 76). Higher-protein or higher-fiber formulations may be useful for supporting satiety and body-condition management, particularly when diet selection is guided by weight trajectory, appetite, energy intake, activity level, and owner adherence (9, 77–79).

These nutritional priorities remain relevant with advancing age, but age-related changes in body composition, energy metabolism, digestive tolerance, urinary physiology, and cognitive function may alter their relative importance and increase the need for closer reassessment and individualized dietary adjustment (21, 80). In aging cats, urine-concentrating ability may decline progressively with age, suggesting that water intake, urine dilution and urinary monitoring may become increasingly relevant nutritional considerations (81). High-moisture diets may support water intake and urinary dilution, particularly in cats (82), while enriched diets containing medium-chain triglycerides, antioxidants, B vitamins, arginine, fish oil and mitochondrial cofactors have been investigated for cognitive support in aging animals (83–85). Given the heterogeneity of aging, precision nutrition in older dogs and cats should move beyond age-based feeding categories toward a functional and response-guided approach to maintaining lean mass, hydration, urinary health, and cognitive function.

### 3.3 Lifestyle, activity and feeding management

Lifestyle and feeding management shape how precision nutrition is implemented in routine veterinary nutrition and home feeding practice. Among these factors, activity level is particularly important because it directly affects energy expenditure and feeding requirements. Working or athletic dogs may require higher energy density and adjustments in macronutrient composition, particularly dietary fat, together with appropriate hydration support, whereas sedentary or indoor pets are more prone to positive energy balance if food intake is not adjusted (86, 87). In cats, indoor lifestyle, diet type, feeding pattern, and treat provision frequency have been associated with overweight or obesity risk, supporting the need to incorporate lifestyle information into individualized feeding plans (6). In cats, increasing dietary water intake through high-moisture diets can increase urine volume and reduce urine specific gravity and RSS for calcium oxalate and struvite, illustrating how dietary adjustment can be guided by urinary endpoints (82). These examples emphasize that precision nutrition must be biologically appropriate as well as practical for routine veterinary care and home feeding. Lifestyle, activity level, diet form and moisture content, hydration, and owner-driven feeding management therefore help determine whether individualized dietary strategies can be implemented consistently and adjusted according to monitored response (21).

## 4 Clinical applications of precision nutrition in dogs and cats

Precision nutrition can be translated into clinical practice when individual variation in disease phenotype and dietary response can be linked to actionable nutritional strategies and measurable outcomes. This section examines four complementary clinical models: obesity, chronic enteropathy, chronic kidney disease, and lower urinary tract disease, with particular emphasis on urolithiasis. Together, they illustrate response-guided nutritional management across body-composition, gastrointestinal, renal-biochemical, and urinary-physicochemical domains. In each context, established clinical measures provide the primary basis for dietary adjustment, while microbiome, omics-derived, and other emerging tools may provide complementary information as their clinical utility becomes better defined. The four representative application models are summarized in Figure 3.

**Figure 3:**
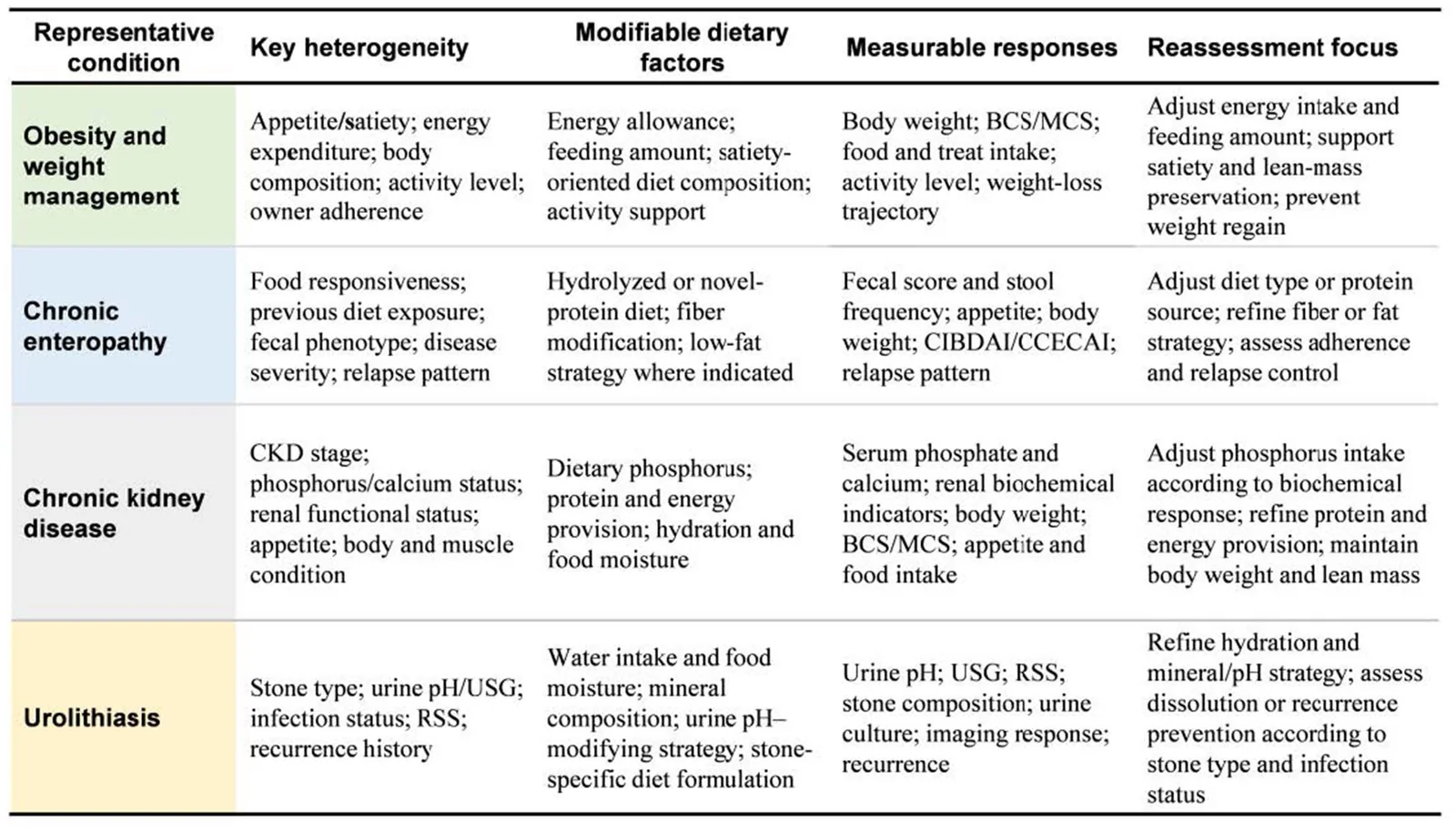
Representative clinical application models of precision nutrition in dogs and cats. Obesity and weight management, chronic enteropathy, chronic kidney disease, and urolithiasis are presented as representative clinical contexts in which individual heterogeneity can be linked to modifiable dietary factors, measurable responses, and longitudinal reassessment. Together, these models illustrate response-guided nutritional management across body-composition, gastrointestinal, renal-biochemical, and urinary-physicochemical domains. BCS, body condition score; CCECAI, canine chronic enteropathy clinical activity index; CIBDAI, canine inflammatory bowel disease activity index; CKD, chronic kidney disease; IRIS, International Renal Interest Society; MCS, muscle condition score; RSS, relative supersaturation; USG, urine specific gravity.

### 4.1 Obesity and weight management

Obesity is one of the most common nutrition-related disorders in companion animals and is associated with reduced quality of life, shortened lifespan, and increased risk of metabolic, orthopedic, respiratory, and cardiovascular comorbidities (75, 76). Established weight-management practice is based on controlled energy restriction, appropriate increases in physical activity, accurate assessment of food and treat intake, and structured long-term follow-up (9, 21). However, individual animals differ in their ability to lose weight and maintain weight loss, reflecting variation in appetite regulation, energy balance, body composition, and owner adherence over time (9, 79). This response heterogeneity highlights the need to consider obesity as more than excess body weight.

At the biological level, companion animal obesity involves interacting genetic, metabolic, and host–microbiome mechanisms that may influence disease risk and dietary response. In dogs, genetic studies have identified appetite and energy-homeostasis pathways implicated in obesity, including POMC deletion, variation in MC4R, and DENND1B-mediated regulation of canine MC4R signaling (16, 52, 53). Metabolomic studies have reported obesity- or weight-loss-associated differences in amino acid, lipid, and L-carnitine-related metabolic profiles in dogs (10, 36). Gut microbiome studies have identified differences in fecal microbial composition and functional features between obese and lean dogs and cats; in dogs, caloric restriction or weight loss may also shift the fecal microbiota toward profiles resembling those observed in lean animals (17, 88, 89). In canine and feline obesity, interactions between gut microbiota and lipid metabolism have also been reported, with possible mechanisms involving microbial metabolites, bile acid metabolism, intestinal barrier function, and appetite regulation (90). Together, these studies highlight appetite regulation, energy and lipid metabolism, and host–microbiome interactions as key biological processes contributing to heterogeneity in obesity and weight-management response.

Nutritional strategies for obesity should be anchored in clinically observable phenotypes and practical measures that can be reassessed during weight management. Energy restriction remains the foundation of weight loss, but diet composition can influence satiety, lean mass preservation, metabolic response, and feeding compliance. Given the involvement of appetite-regulatory pathways in obesity, satiety-oriented feeding represents a biologically plausible and clinically applicable strategy: a high-protein, high-fiber diet has been shown to improve satiety and reduce voluntary food intake in dogs (77), although these effects have not been demonstrated to be genotype-specific. Fermentable fibers such as beet pulp, inulin and fructo-oligosaccharides may support satiety and microbial fermentation (78). The metabolic findings involving lipid and carnitine pathways also provide a rationale for investigating metabolically targeted formulations, but intervention evidence remains more limited. A high-protein, high-fiber diet enriched with antioxidants and L-carnitine has been evaluated in cats after spaying, with the study primarily addressing prevention of post-neutering weight gain rather than treatment of established obesity (91). In addition, choline altered circulating lipid and lipoprotein concentrations without improving body composition or energy expenditure during weight maintenance in lean and obese male cats (92). Therefore, current evidence supports a role for metabolomic profiles primarily in metabolic phenotyping and response assessment, rather than in guiding the selection of specific supplements or dietary formulations.

Microbiome-targeted approaches represent an emerging area of obesity research in companion animals. In obese dogs, probiotic supplementation has been associated with changes in body weight, gut microbiome composition, and metabolic profiles (93), providing intervention-level evidence that selected microbial targets may influence obesity-related outcomes. However, these effects remain strain- and study-specific, and current evidence is insufficient to support routine selection of probiotics according to an individual baseline microbiome profile. A canine-derived *Ligilactobacillus animalis* strain has shown anti-obesity potential in a mouse model of diet-induced obesity, but its efficacy and safety in obese dogs or cats remain to be validated (94). Similarly, *Akkermansia muciniphila* may represent a candidate taxon for further investigation in companion animal obesity, although current evidence in dogs and cats is insufficient to determine its role in obesity or whether dietary intervention can consistently alter its abundance or activity (95). Thus, probiotic and microbiome-targeted strategies may become useful components of precision weight management if their effects can be linked to defined phenotypes, dietary context, and measurable metabolic or clinical changes.

In practice, precision nutrition for obesity should be based on repeated assessment of body weight, body condition score, muscle condition score, food and treat intake, activity level, and weight-loss trajectory, with dietary adjustments made according to the observed response. At present, energy restriction, feeding-amount adjustment, satiety-oriented diet selection, and structured monitoring are the most directly actionable strategies. Genetic information may help refine obesity-risk and appetite-related assessment, metabolomic profiles may support metabolic phenotyping and response monitoring, and microbiome-targeted interventions represent an emerging adjunctive approach. Their broader incorporation into routine dietary decision-making will require evidence that they improve diet selection or response prediction beyond established clinical assessment.

### 4.2 Chronic enteropathy and dietary management

Chronic enteropathy (CE) refers to a heterogeneous group of chronic or recurrent gastrointestinal disorders in dogs and cats, typically characterized by persistent or recurrent clinical signs such as diarrhea, vomiting, weight loss, and hyporexia (96, 97). In dogs, recent guidance from the American College of Veterinary Internal Medicine (ACVIM) guidance uses chronic inflammatory enteropathy (CIE) as the preferred terminology and places response to treatment trials at the center of clinical phenotyping. Dietary treatment trials are recommended as the first-choice diagnostic intervention in clinically stable dogs, with 38%−89% of affected dogs reported to be food-responsive; immunomodulator-responsive disease is considered after adequate dietary trials, whereas empirical antimicrobial treatment is no longer recommended (96). Recent frameworks have proposed replacing the traditional antibiotic-responsive enteropathy category with microbiota-related modulation-responsive enteropathy (MrMRE), reflecting a shift toward microbiota-directed management rather than empirical antibiotic treatment (98). Within a precision-nutrition framework, this clinical heterogeneity and response-based classification highlight the need to consider current clinical presentation together with dietary history, fecal characteristics, diet tolerance, relapse pattern, and responses to previous interventions (28, 99).

The pathophysiology of CE involves diet–microbiome–host interactions, including altered epithelial barrier function, mucosal immune activation, and microbial metabolism (15, 28). Dysbiosis and altered microbial fermentation have been reported in dogs with CE, including changes in fecal microbiota structure and short-chain fatty acid concentrations (37, 46). Importantly, some of these mechanisms have been linked directly to dietary response: remission induced by a hydrolyzed protein diet was accompanied by reduced dysbiosis and increased secondary bile acids, including lithocholic and deoxycholic acids (18). This supports the potential use of microbial and bile-acid changes as response-related biomarkers, although they are not yet validated for prospectively selecting a specific diet. The fecal dysbiosis index provides a more targeted measure of microbial disruption, but current evidence supports its use primarily as complementary information rather than as a stand-alone predictor of dietary response (96). Similarly, although impaired barrier function is biologically relevant to CIE, candidate barrier biomarkers remain immature; for example, serum zonulin concentrations did not differ significantly between dogs with CE and healthy controls in a prospective study (100). Thus, microbiome, bile-acid, short-chain fatty acids (SCFAs), tryptophan-related, and barrier markers currently have greater value for mechanistic characterization and response assessment than for stand-alone dietary prescription.

Therapeutic diets remain the most clinically established nutritional intervention and should be prioritized according to dietary history, gastrointestinal and fecal phenotype, and relevant clinical findings. Hydrolyzed or novel-protein diets are appropriate options when adverse responses to dietary proteins are suspected, whereas fiber-enriched strategies have stronger clinical relevance for large-bowel or fiber-responsive phenotypes; low-fat diets are particularly relevant in selected dogs with protein-losing enteropathy or intestinal lymphangiectasia (96). Soluble and fermentable fibers may improve stool consistency through effects on intestinal water balance and microbial fermentation. In dogs with chronic large-bowel diarrhea, a fiber-enriched therapeutic diet improved stool quality while shifting microbial metabolism toward saccharolytic fermentation and increasing tryptophan-derived indole metabolites (101, 102), providing a direct example of how dietary intervention can modify both the clinical phenotype and microbiome-related responses. A study in healthy dogs showed that fructo-oligosaccharides altered fecal fermentation products and bacterial populations (103), although its relevance to CIE remains mechanistic rather than clinically established. Current evidence supports considering prebiotics, probiotics and synbiotics mainly as adjunctive options in dogs showing an incomplete response to dietary trials alone, rather than as substitutes for dietary treatment (96). Clinical effects are also likely to be product- and strain-specific, and several controlled studies have not demonstrated additional clinical benefit when probiotic products were added to an otherwise effective elimination diet (96). Accordingly, probiotics and synbiotics may presently be considered promising adjunctive tools, whereas postbiotic and other immune- or barrier-targeted nutritional approaches remain more exploratory until their added clinical benefit is demonstrated.

In dogs, a precision-nutrition diet should be predefined and response-guided. A therapeutic diet should be fed exclusively for at least 2 weeks, with response assessed regularly using Canine inflammatory bowel disease activity index (CIBDAI) or Canine chronic enteropathy clinical activity index (CCECAI), together with stool quality, appetite, body weight, and dietary adherence. When response remains inadequate despite good adherence, alternative diets should be considered before immunomodulatory treatment; current canine CIE guidance recommends at least three well-conducted dietary trials when appropriate, with an effective diet maintained for at least 12 weeks after remission (96). Because these operational criteria are derived primarily from canine CIE guidance, dietary management in cats should be individualized according to clinical phenotype, diet tolerance and longitudinal response. In this framework, changes in fecal phenotype, appetite, body weight, and relapse pattern can directly trigger dietary reassessment, whereas dysbiosis indices, bile acids, SCFAs, and metabolomic profiles currently provide supportive rather than prescriptive information. Therefore, in CE/CIE, precision nutrition should integrate phenotype-informed diet selection with predefined response criteria, repeated reassessment, and iterative dietary adjustment, while microbiome- and metabolite-derived markers currently remain supportive rather than prescriptive.

### 4.3 Chronic kidney disease and response-guided nutritional management

Chronic kidney disease (CKD) is defined by structural and/or functional abnormalities of one or both kidneys that persist for at least 3 months and may be accompanied by impairment of renal excretory, metabolic, and endocrine functions (104, 105). A key nutrition-related consequence is disruption of phosphate homeostasis (106, 107). As glomerular filtration rate declines, renal phosphate excretion becomes increasingly limited, while fibroblast growth factor 23 (FGF23) rises as an early compensatory response to enhance phosphate excretion and suppress calcitriol formation (107). These adaptations can initially maintain serum phosphorus despite declining renal function, but with disease progression, impaired FGF23–Klotho signaling, increasing parathyroid hormone activity, and reduced calcitriol contribute to calcium–phosphorus imbalance and CKD–mineral and bone disorder (CKD-MBD) (106, 107). In cats, FGF23 may increase before overt hyperphosphatemia and has been associated with CKD progression and survival (106, 107).

The gut–kidney axis represents an additional nutrition-sensitive mechanism. Dietary tryptophan, tyrosine, carnitine, and related nutrients can enter microbial–host co-metabolic pathways that contribute to the production of gut-derived uremic solutes, including indoxyl sulfate (IS), p-cresyl sulfate (pCS), and trimethylamine-N-oxide (108). Cats with CKD show reduced fecal microbial richness and increased circulating IS, while metabolomic studies have identified disturbances in amino acid and carnitine metabolism together with accumulation of several uremic metabolites (108, 109). Targeted bile acid metabolomics has further identified reduced fecal ursodeoxycholic acid and a distinct secondary-bile-acid dysmetabolism phenotype in a subset of cats with CKD (110). IS has also been associated with circulating FGF23 in feline CKD, suggesting a potential interaction between microbial-derived metabolic burden and disturbances in mineral metabolism (107, 111). These findings position gut microbial metabolism as an additional and potentially modifiable component of CKD heterogeneity, alongside the dominant influence of declining renal clearance on circulating uremic solutes.

In current clinical practice, dietary management follows the stage-based CKD recommendations of the International Renal Interest Society (IRIS) (112). These recommendations also address hydration, proteinuria, blood pressure, electrolyte and acid–base disturbances, and other disease-specific complications (105, 112). Controlled clinical trials in dogs and cats with naturally occurring CKD have shown that CKD-targeted dietary modification reduces uremic complications and improves survival-related outcomes (113, 114). Within this framework, therapeutic priorities vary with CKD stage. In stages 1 and 2, management focuses primarily on slowing disease progression and preserving remaining kidney function, whereas from stage 3 onward, increasing emphasis is placed on controlling extra-renal clinical signs and maintaining quality of life (112). Maintenance of nutritional status is also important; body weight, body and muscle condition, appetite, and energy intake are routinely assessed, and dietary strategies supporting adequate energy and essential amino acid intake can help preserve body weight and lean mass in cats with early CKD (5, 115).

Precision nutrition in CKD involves individualized adjustment of established nutritional strategies according to disease stage, biochemical profile, nutritional status, and longitudinal response. Phosphorus restriction is an established nutritional strategy in CKD, with the degree of restriction guided by serum phosphate and concurrent monitoring of calcium; intestinal phosphate binders may be introduced when dietary measures alone are insufficient to achieve stage-specific serum phosphate targets (5, 112). In cats developing ionized hypercalcemia during marked phosphorus restriction, attenuation of restriction can normalize ionized calcium while maintaining phosphorus control, illustrating how biochemical response can directly guide dietary adjustment (116). FGF23 may provide additional insight into phosphate regulation in selected cats, particularly when serum phosphorus remains within the recommended range for the corresponding CKD stage (107, 117). Protein and energy intake should be individualized according to clinical and nutritional status, with emphasis on maintaining appetite, body weight, and lean body mass (5). Within this response-guided framework, emerging evidence may further refine nutritional management by considering dietary and metabolic characteristics beyond conventional biochemical measures. Experimental studies in healthy cats indicate that phosphorus source, solubility, and Ca:P ratio influence renal phosphorus excretion and postprandial phosphorus and parathyroid hormone responses, while prolonged exposure to high inorganic phosphorus combined with a low Ca:P ratio has been associated with renal functional and structural changes (118–120). In parallel, fermentable fibers and other gut-directed nutritional strategies can modify microbial-derived metabolites, and recent multi-omics studies have identified substantial inter-individual variation in metabolic responses to microbiome-directed interventions (121–123). Together, these findings extend CKD precision nutrition from established stage- and biomarker-guided adjustment toward more detailed characterization of dietary exposure and metabolic response phenotypes.

### 4.4 Lower urinary tract disease: response-guided management of urolithiasis

Lower urinary tract signs in dogs and cats may arise from different underlying disorders. In cats, feline lower urinary tract disease encompasses conditions presenting with lower urinary tract signs, including feline idiopathic cystitis, urolithiasis, and urinary tract infection, as well as less common causes such as neoplasia, trauma, and congenital or acquired anatomical abnormalities; urethral obstruction may occur as a potentially life-threatening manifestation of several of these disorders (124). Among these conditions, urolithiasis is particularly suited to a precision-nutrition framework because stone composition, urine pH, urine specific gravity, urine culture, RSS where available, imaging findings, and recurrence history can directly guide and monitor dietary management (27, 125).

Urolithiasis is multifactorial and is strongly influenced by the urinary physicochemical environment. Urine concentration, pH, lithogenic solute excretion, crystallization modifiers, infection status, and host susceptibility contribute to crystal formation, while several of these factors can be modified through water intake and dietary composition (27, 126). RSS integrates urinary solute concentrations and pH to estimate mineral-specific crystallization potential and can therefore be used to assess dietary effects; although it should be interpreted together with stone type and other urinary and clinical findings (125, 127). A clinically important distinction is that struvite urolithiasis in dogs is often infection-associated, whereas sterile struvite uroliths in cats are commonly managed through dietary dissolution (128, 129). Infection-associated struvite formation is typically linked to urease-producing bacteria, such as *Staphylococcus* spp. and *Proteus* spp., which hydrolyze urea, increase urinary ammonia and pH, and promote magnesium ammonium phosphate crystallization (130). Therefore, dietary management of struvite urolithiasis should account for infection status, because dietary modification is unlikely to be effective unless the underlying bacterial infection is controlled. In cats, sterile struvite uroliths are often amenable to dissolution using magnesium-restricted, moderately acidifying diets that promote urine dilution, with monitoring of urine pH and radiographic response (27, 128, 129). In contrast, calcium oxalate urolithiasis cannot be dissolved nutritionally and is managed primarily through recurrence prevention, including increasing urine volume, avoiding excessive urinary acidification, moderating mineral and oxalate-related risk factors, and monitoring RSS where available (125). High-moisture diets provide a practical example of precision-oriented dietary adjustment in cats because increasing dietary water intake can increase urine volume and reduce urine specific gravity and relative supersaturation for calcium oxalate and struvite (82, 125). Thus, stone composition and urinary physicochemical phenotype already support different, clinically established nutritional pathways rather than a uniform “urinary diet” approach.

Microbiome and metabolomic studies may provide additional insight into urinary risk phenotypes and future nutrition-related interventions. Studies have linked calcium oxalate urolithiasis with alterations in the gut and urinary microbiota, including changes in oxalate-degrading bacteria and urinary microbial communities (131–133). Urinary metabolomic profiling has also identified candidate biomarkers that may help distinguish calcium oxalate from struvite urolithiasis in dogs, including metabolites involved in phenylalanine, nicotinate and nicotinamide, tryptophan and glycerophospholipid metabolism (134). Microbiota-targeted strategies are being investigated, and selected strains of *Lactobacillus, Bifidobacterium* and *Bacillus subtilis* have shown oxalate-degrading or anti-crystallization potential *in vitro* or in experimental settings (135–138). These findings may support future microbiome- or metabolite-informed assessment of urinary risk, but their clinical value will depend on whether they improve recurrence-risk evaluation or dietary adjustment when interpreted alongside established urinary measures. Similarly, succinate-related pathways may provide mechanistic insight into calcium deposition and crystal–cell interactions, but the safety, efficacy, and dietary relevance of succinate-based interventions in dogs and cats remain to be established (139). For infection-related urinary disorders, cranberry extracts and D-mannose may serve as complementary anti-adhesion strategies, although they are more relevant to bacterial adherence and urinary tract infection risk than to direct control of mineral crystallization (140, 141).

In urolithiasis, precision nutrition should translate stone composition and urinary risk profiles into targeted dietary prevention and management. Currently actionable decisions include selection of dissolution vs. recurrence-prevention strategies, modification of water intake and food moisture, and adjustment of mineral or urinary pH targets according to stone type, infection status, and urinary response. Urinary diets and related nutritional strategies should be selected and adjusted according to stone type, urinary physicochemical response, and infection status. This approach helps distinguish dissolution-oriented management for sterile struvite from recurrence-prevention strategies for calcium oxalate, while linking dietary adjustment to urinary endpoints such as urine dilution, urinary pH and relative supersaturation. Less common urolith types, including urate, xanthine and cystine, also require stone-specific nutritional management. Urate and xanthine prevention generally emphasizes increased urine dilution and control of dietary purine intake, whereas cystine management may include increased water intake, urinary alkalinization and modification of dietary protein intake, with strategies tailored to the underlying metabolic or genetic disorder (27, 126). Beyond these established measures, microbiome and metabolomic profiles may eventually provide complementary information for refining urolith risk assessment and monitoring dietary response, while microbiota-targeted interventions remain exploratory and require clinical validation before being incorporated into nutritional management. Thus, precision nutrition in urolithiasis should be viewed as a dynamic, endpoint-guided framework linking diet composition, hydration strategy, and longitudinal urinary response.

### 4.5 Additional clinical applications of precision nutrition

Although the clinical models discussed above focus on obesity, chronic enteropathy, chronic kidney disease, and urolithiasis, the same response-guided framework may also be relevant to other nutrition-related conditions in dogs and cats. Diabetes mellitus represents a metabolic condition in which dietary composition, body condition, and energy intake are individualized together with insulin therapy and glycemic monitoring (142). Dermatological disorders and adverse food reactions provide a different application, where elimination or hydrolyzed-protein diets can be evaluated through clinical response, while nutritional support for atopic disease is usually integrated with broader dermatological management (143). Hepatobiliary and pancreatic disorders require more disease-specific strategies because energy, protein, fat, and selected nutrient modifications depend strongly on the underlying diagnosis, disease severity, and species-specific physiology (144, 145). Musculoskeletal disease and cognitive dysfunction are primarily managed through functional outcomes, including body-weight control, mobility, behavior and quality of life, with omega-3 fatty acids and nutrient-enriched dietary strategies providing supportive options (85, 146, 147). Oncology similarly emphasizes maintenance of energy intake, body weight, and lean mass as part of supportive nutritional care (148).

However, across many of these conditions, individualized nutritional management is still driven mainly by disease-specific clinical assessment, therapeutic nutrition, and functional outcomes. Validated nutritional biomarkers that can prospectively guide diet selection or define individualized adjustment thresholds remain limited, and dietary effects are often difficult to separate from concurrent medical treatment, disease severity, and comorbidities. These areas therefore represent important opportunities for extending precision nutrition as disease-specific phenotyping, response markers, and longitudinal nutritional evidence become better defined.

## 5 Future perspectives and translational challenges

### 5.1 From biomarker discovery to clinically useful evidence

The further development of precision nutrition in companion animals will depend on demonstrating that candidate biomarkers and data-driven approaches can add value to established nutritional assessment and support more individualized dietary decisions. Within a precision-nutrition workflow, biomarkers can serve distinct roles: diagnostic biomarkers support baseline disease or phenotype classification, predictive biomarkers help identify likely responders and inform dietary selection before intervention, and monitoring biomarkers track response, relapse, and the need for adjustment during follow-up (149). At present, companion-animal precision nutrition is closest to clinical implementation through monitoring and response-related measures rather than validated molecular predictive biomarkers. Body weight, body condition score and muscle condition score in obesity, fecal characteristics and CIBDAI/CCECAI in canine CIE, and urine pH, urine specific gravity, stone composition and relative supersaturation in urolithiasis can already be linked to reassessment or dietary adjustment (21, 27, 96). In contrast, POMC variation, metabolomic profiles, microbiome and bile-acid changes, and fecal calprotectin provide useful information on susceptibility, metabolic phenotype, or treatment response, but have not yet been validated to select a specific diet or define thresholds for dietary adjustment (10, 16, 18, 96, 150). Thus, monitoring and response-related measures currently have greater practical utility than omics-derived predictive biomarkers.

Future studies should clarify which biomarkers and data-derived features provide information that is useful for nutritional decision-making, rather than simply identifying signals associated with biological variation or disease status. Cross-sectional studies are valuable for biomarker discovery, but prospective and longitudinal designs are required to determine whether baseline markers predict subsequent dietary response or whether longitudinal changes reliably reflect treatment response. Therefore, predictive biomarkers should be prospectively validated for their ability to anticipate dietary response using clearly defined clinical and nutritional outcomes and should provide information beyond conventional clinical assessment (149, 151). Generating such evidence is particularly challenging in companion animals because recruitment, retention and repeated sampling can be difficult. Animal-welfare considerations favor minimally invasive samples such as blood, urine and feces, while tissue collection is generally more restricted (48, 152). Species-specific *in vitro* models, including canine and feline organoids, may complement these studies by supporting mechanistic validation and preliminary screening of candidate biomarkers or nutritional interventions (153–155). Such models may help prioritize candidate biomarkers, nutritional pathways, and interventions for subsequent *in vivo* evaluation, but they should be regarded as mechanistic validation tools rather than substitutes for longitudinal dietary intervention studies. Accordingly, future biomarker development in companion animals should emphasize clinically interpretable and repeatable measures that can be evaluated longitudinally, while tissue-based and *in vitro* approaches should serve as complementary tools for mechanistic validation and candidate prioritization.

### 5.2 Data standardization, multi-omics integration and AI decision support

Precision nutrition requires data that are standardized across studies, interoperable across analytical platforms, and interpretable in clinical nutritional practice. Current companion-animal studies often differ in sample collection, analytical workflows, phenotype definitions, and outcome measures. These methodological differences can materially affect the biological signals detected. For example, a comparison of canine fecal metagenomic workflows showed substantial effects of DNA extraction, library preparation, and bioinformatic approaches on microbial community profiles, while matched canine metabolomic analyses demonstrated that serum, plasma, and urine are not interchangeable matrices and differ in their ability to capture diet-associated variation (156, 157). To improve comparability across studies, standardized data collection should include consistent host, dietary, clinical, sampling, and longitudinal-response information in addition to analytical procedures. Large longitudinal canine cohorts illustrate how such structured biospecimen and metadata collection can be implemented at scale (158). Multi-omics approaches have begun to integrate complementary data layers, such as microbiome and metabolite profiles, to characterize diet-responsive changes in dogs (159); however, their use for prospective prediction of individual dietary response remains exploratory and will require larger longitudinal datasets and independent validation.

Artificial intelligence and machine-learning approaches may support precision nutrition by integrating clinical phenotypes, dietary information, omics-derived features, and longitudinal data generated by digital monitoring tools. In companion animals, current applications are mainly focused on data integration, pattern recognition, and response monitoring: for example, machine-learning analysis of longitudinal microbiome and metabolite data has distinguished dietary treatment groups in dogs (159), while smart feeders, home scales, and activity monitors have been incorporated into weight-management programs in client-owned cats (160). However, the translation of digital data into nutritional decisions requires additional validation; a commercial canine activity monitor, for example, showed limited accuracy for estimating daily energy requirements (161). Thus, device accuracy, data completeness, owner engagement, model interpretability, independent validation, and professional oversight remain important considerations when AI-derived outputs are used to support nutritional assessment and dietary decisions, within an evolving regulatory landscape for veterinary AI (162, 163).

Beyond analysis and monitoring, AI may eventually contribute to prospective intervention prioritization. In human medicine, patient-specific digital twins have been used to simulate dose-dependent drug responses and subsequently compare these predictions with clinical outcomes, while AI-based digital-twin systems in precision nutrition have integrated continuous physiological and dietary data to predict metabolic responses and dynamically refine dietary recommendations (164, 165). AI-accelerated virtual screening in drug development further illustrates how computational models can narrow a large candidate space before experimental validation (166). Accordingly, AI in companion-animal precision nutrition is best positioned as a decision-support tool for prioritizing and refining nutritional strategies, with final dietary decisions anchored in validated evidence and longitudinal clinical response.

### 5.3 Translating precision nutrition into practice

The translation of precision nutrition into practice requires dietary strategies that are scientifically justified, clinically feasible, and compatible with owner-driven feeding management. Nutritional management should integrate life stage, body condition, activity level, dietary exposure, owner-driven feeding practice, and relevant clinical findings, with subsequent reassessment based on the observed response. For example, in a neutered indoor cat showing progressive weight gain, energy allowance and feeding strategy can be refined according to body condition, actual food and treat intake, activity and household feeding practices, with subsequent weight trajectory used to determine whether further adjustment is needed (21, 73). When disease-specific nutrition is required, the same principle applies: dietary selection is informed by the dominant clinical phenotype and then revised according to measurable response, as illustrated by dietary trials in CIE and stone-specific management in urolithiasis (27, 96). These examples illustrate how precision nutrition can already operate as a response-guided approach to nutritional management rather than simply the selection of a specialized diet.

In this review, precision tools are grouped according to their current clinical applicability. Established tools can already support diet selection, adjustment, or monitoring in routine practice; supportive or emerging tools, including biomarkers and microbiome or metabolomic profiles, may refine phenotyping or response assessment but are not yet sufficient for independent diet selection; and multi-omics- or AI-based prediction systems remain exploratory and require further clinical validation (149–151, 159). Digital technologies may also support longitudinal monitoring, with smart feeders, home scales and activity monitors already incorporated into weight-management programs in client-owned cats (160). Their clinical value will ultimately depend on whether the information generated contributes meaningfully to nutritional assessment and adjustment. Therefore, a realistic implementation pathway is to anchor current practice in established clinical and dietary measures, progressively incorporate validated supportive tools, and reserve advanced predictive approaches for settings in which they demonstrate clear added value. This staged approach also provides a basis for evidence-based clinical recommendations and realistic applications in pet-food development. As longitudinal evidence accumulates, this framework may also support earlier, risk-informed nutritional management across the life course, provided that its predictive and clinical value is prospectively validated.

## 6 Conclusion

Precision nutrition provides a data-driven and response-guided framework for extending population-level feeding recommendations toward individualized nutritional management in companion animals. This approach accounts for individual variation in nutritional requirements, disease risk, and dietary response through the combined interpretation of host-related determinants, clinical phenotypes, dietary history, omics-derived information, and longitudinal monitoring. In dogs and cats, precision nutrition is currently most clinically applicable when dietary strategies can be linked to measurable endpoints, such as body condition, fecal quality, urinary parameters, stone composition and monitored dietary response. Emerging omics-derived biomarkers, digital monitoring tools and algorithm-assisted approaches may further refine dietary assessment and intervention, but they require validation before routine use in dietary decision-making. Future progress will depend on standardized data collection, validated biomarkers, controlled feeding trials, longitudinal cohorts, interpretable algorithms, and real-world outcome monitoring. Ultimately, precision nutrition in companion animals is best understood as an evidence-based and iterative nutritional decision framework rather than simple diet customization. The value of this framework will ultimately depend on its ability to improve dietary management and generate measurable benefits for animal health and long-term disease control.
